# Mix and Match: Phenotypic Coexistence as a Key Facilitator of Cancer Invasion

**DOI:** 10.1007/s11538-019-00675-0

**Published:** 2020-01-17

**Authors:** Maximilian A. R. Strobl, Andrew L. Krause, Mehdi Damaghi, Robert Gillies, Alexander R. A. Anderson, Philip K. Maini

**Affiliations:** 1 Wolfson Centre for Mathematical Biology, Mathematical Institute, University of Oxford, Radcliffe Observatory Quarter, OX2 6GG Oxford, UK; 2 Department of Cancer Physiology, Moffitt Cancer Center, Magnolia Drive, Tampa, 12902 USA; 3 Department of Integrated Mathematical Oncology, Moffitt Cancer Center, Magnolia Drive, Tampa, 12902 USA

**Keywords:** Tumour invasion, Cooperation, Travelling waves, Coexistence theory, Reaction–diffusion

## Abstract

Invasion of healthy tissue is a defining feature of malignant tumours. Traditionally, invasion is thought to be driven by cells that have acquired all the necessary traits to overcome the range of biological and physical defences employed by the body. However, in light of the ever-increasing evidence for geno- and phenotypic intra-tumour heterogeneity, an alternative hypothesis presents itself: could invasion be driven by a collection of cells with distinct traits that together facilitate the invasion process? In this paper, we use a mathematical model to assess the feasibility of this hypothesis in the context of acid-mediated invasion. We assume tumour expansion is obstructed by stroma which inhibits growth and extra-cellular matrix (ECM) which blocks cancer cell movement. Further, we assume that there are two types of cancer cells: (i) a glycolytic phenotype which produces acid that kills stromal cells and (ii) a matrix-degrading phenotype that locally remodels the ECM. We extend the Gatenby–Gawlinski reaction–diffusion model to derive a system of five coupled reaction–diffusion equations to describe the resulting invasion process. We characterise the spatially homogeneous steady states and carry out a simulation study in one spatial dimension to determine how the tumour develops as we vary the strength of competition between the two phenotypes. We find that overall tumour growth is most extensive when both cell types can stably coexist, since this allows the cells to locally mix and benefit most from the combination of traits. In contrast, when inter-species competition exceeds intra-species competition the populations spatially separate and invasion arrests either: (i) rapidly (matrix-degraders dominate) or (ii) slowly (acid-producers dominate). Overall, our work demonstrates that the spatial and ecological relationship between a heterogeneous population of tumour cells is a key factor in determining their ability to cooperate. Specifically, we predict that tumours in which different phenotypes coexist stably are more invasive than tumours in which phenotypes are spatially separated.

## Introduction

Tissue invasion is a hallmark of cancer (Hanahan and Weinberg 2000). If a tumour is detected before it has started to spread into the surrounding tissue; then, the tumour is termed *benign*, and the chances of survival are high. If the tumour has started to spread, breaching the basement membrane, survival rates are significantly decreased and the tumour is termed *malignant* (“badly born”). In 90% of patients, the cause of death is not the primary tumour, but the disruption of normal body function caused by metastatic disease (Sporn 1996)—for which invasion is the first critical step.

Due to the profound damage caused by the uncontrolled spread of cells, a great number of mechanisms have evolved to ensure that cells—even those that might have started to escape homeostatic control—remain localised. One important barrier, for example, is the extra-cellular matrix (ECM), a dense mixture of proteins encapsulating the cells in healthy tissue (Stetler-Stevenson et al. 1993; Werb 1997). The proteins in the ECM form a strong scaffolding which physically anchors tissue cells in place and activates intra-cellular signalling pathways which suppress cell movement and regulate proliferation (Stetler-Stevenson et al. 1993; Werb 1997; McKinnell 1998; Werb 1997; Bloom and Zaman 2014). A further important barrier to local expansion of the tumour is the inhibitory environment created by the healthy tissue (stroma) surrounding the tumour. For example, an analysis of 432 different cancer–fibroblast co-cultures found that 41% of the investigated pairings led to reduced cancer growth (Wadlow et al. 2009).

Research over the past decades has elucidated in great detail the molecular mechanisms used by cancer cells to overcome these barriers. In order to remodel or degrade the ECM, tumour cells use matrix-degrading enzymes (MDEs) such as matrix metalloproteinases (Stetler-Stevenson et al. 1993; Curran and Murray 1999; Hanahan and Weinberg 2000). Similarly, in order to overcome the growth inhibition from the surrounding stroma, tumour cells can coerce healthy cells into tumour-promoting phenotypes (e.g. tumour-associated fibroblasts) or eradicate them. In a series of papers, Gatenby and co-workers have proposed that an important contribution to this transformation is the acidification of the tissue environment by the tumour, a theory known as the “acid-mediated invasion hypothesis” (Gatenby and Gawlinski 1996; Gatenby and Gillies 2004; Gatenby et al. 2006; Gillies et al. 2008). Many invasive cancers are characterised by their use of glycolysis for energy generation even in conditions under which more efficient aerobic respiration would be feasible, a paradox known as the “Warburg effect” (Warburg and Dickens 1930; Gillies et al. 2008). Gatenby and co-workers argue that the acidification due to upregulated glycolysis, which ranges over 0.5–1 pH units (Wike-Hooley et al. 1984; Helmlinger et al. 1997), results in death of normal cells, thereby allowing tumour cells to expand (Gatenby and Gawlinski 1996; Gatenby and Gillies 2004; Gatenby et al. 2006; Gillies et al. 2008). This hypothesis is supported, for example, by experiments showing that low pH leads to increased rates of cell death (Tannock and Rotin 1989) or that administration of a neutralising buffer can reduce tumour expansion in mice (Ibrahim Hashim et al. 2011).

The advances in our molecular understanding of invasion have been accompanied by a significant body of theoretical work that has aimed to integrate the insights from different spatial and temporal scales to identify clinical implications and to guide future experiments (see Araujo and McElwain 2004 for an excellent review). Gatenby and Gawlinski developed a mathematical model based on reaction–diffusion equations to investigate the feasibility and implications of the acid-mediated invasion hypothesis (Gatenby and Gawlinski 1996). In their three-compartment model, the authors represent tissue as a mixture of healthy stromal cells, cancer cells, and acid released by the tumour cells (Gatenby and Gawlinski 1996). They identify different modes of invasion depending on the system parameters and predict that particularly aggressive invasion gives rise to a gap between the advancing tumour and retreating tissue front (Gatenby and Gawlinski 1996). Subsequent work has more formally analysed this model and suggested new experiments that could be used to test the underlying assumptions (Fasano et al. 2009; McGillen et al. 2014).

In addition to the role of acid, the dynamics of ECM remodelling and degradation has also been studied. Considering the ECM as a purely physical barrier, Martin and co-workers (Martin et al. 2010) used an extension of the Gatenby–Gawlinski model to demonstrate that if a collaboration between the tumour cells and the stroma is required to degrade the matrix, then highly acidic tumours may be encapsulated and unable to invade. Other studies instead considered the stimulatory effects that certain by-products of matrix degradation have on activation and direction of tumour cell movement. Anderson et al. (2000) showed in a partial differential equation (PDE) model that such an ECM gradient driven migration (haptotaxis) can influence the shape of the growing tumour. In a series of papers, the group led by Mark Chaplain have further characterised the importance of cell–cell adhesion in tumour invasion (Byrne and Chaplain 1996; Gerisch and Chaplain 2008; Domschke et al. 2014) and identified the plasminogen urokinase activation system as a key driver of invasion (Chaplain and Lolas 2005; Ramis-Conde et al. 2008; Andasari et al. 2011).

**Fig. 1:**
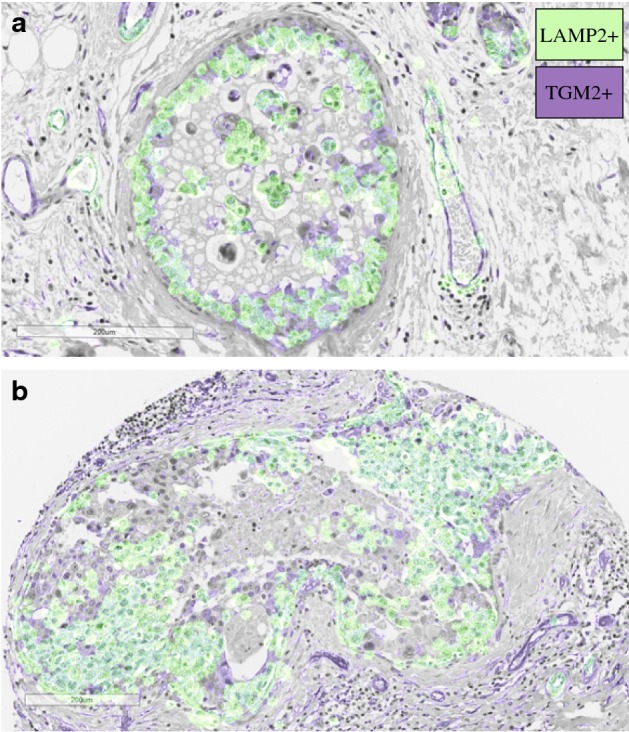
Areas of acid production and matrix remodelling in human breast cancer ducts. Acid production was defined by expression of the acid adaptation marker LAMP2 (green). Matrix remodelling was defined by expression of TGM2 (purple). For visualisation purposes, masks were extracted and overlaid on a haematoxylin and eosin stain of the same tissue (see Section A1 for details). **a** Example of a ductal carcinoma in situ that has not yet invaded the surrounding tissue. **b** Example of an invasive cancer that has breached the duct. We observe that not all cells are expressing LAMP2 or TGM2. Could there be cooperation between cells with different traits? (Color figure online)

While we have an increasing understanding of *how* tumour cells invade, an important open question remains as to *when in oncogenesis* invasion emerges. Traditionally, invasion is thought to be carried out by a subset of cancer cells that have acquired all the necessary traits to overcome the host’s various defence mechanisms. However, over the past decade, it has become clear that tumours are a heterogeneous mixture of cells that differ in their genetic make-up and phenotypic behaviour (Merlo et al. 2006; Gerlinger et al. 2012; Basanta and Anderson 2013). As part of a recent study, currently in preparation for publication (Damaghi et al. 2019), we observed significant heterogeneity in the distribution of matrix remodelling activity and acid adaptation among cancer cells in human breast cancer ducts (Fig. 1). Even along the invasive front, the overlap of the regions of acid production and matrix remodelling is not complete (Fig. 1b). While further experimental work will be required to ratify these observations, they led us to ask the question: instead of being driven by group of “super-cells”, could cancer invasion rather be an emergent property of cooperating specialist cells?

There is mounting evidence for cooperation among tumour cells (Axelrod et al. 2006; Archetti and Pienta 2018). A Wnt1-driven mouse model of breast cancer, for example, has been shown to be composed of two cell types: one expressing Wnt1 and the other expressing the associated Lrp5 receptor (Kim et al. 2011; Cleary et al. 2014). Interaction with the other cell type allows each population to grow faster and drives tumour growth (Kim et al. 2011; Cleary et al. 2014). Alternatively, production of diffusible growth factors can allow for cross-feeding among tumour cells, where a cell produces one type of growth factor and receives the others from its neighbours (Axelrod et al. 2006; Archetti and Pienta 2018). Given that cells have been shown to support each others’ growth, it seems plausible that they may also cooperate to overcome the body’s defences during tissue invasion.

The aim of this paper is to use a mathematical model to investigate the feasibility and the implications of this hypothesis in the context of acid-mediated invasion. We will extend the Gatenby–Gawlinski model so that it includes obstruction both from the stroma and the ECM. Specifically, we will assume that stromal cells suppress growth, while the ECM blocks cell movement. Unlike previous work (Ramis-Conde et al. 2008; Martin et al. 2010), we will assume that no single tumour cell can remove both obstructions. Instead, we will assume that there are two cancer phenotypes: (i) an acid-producing phenotype which removes stroma and (ii) an ECM-degrading phenotype. We will assume that these distinct phenotypes cooperate to remove obstructions, but must also compete with one another for resources. Through linear stability analysis and numerical simulations of the resulting system of five differential equations, we will study under which circumstances a mixture of the two populations (as defined by the relative inter-species competition) develops into an invasive cancer. The images are available upon reasonable request from the corresponding author.

**Fig. 2:**
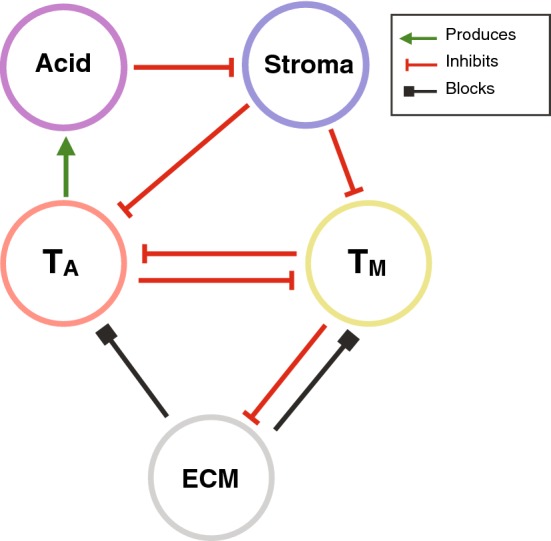
Interaction diagram of our model. Stroma inhibits tumour cell proliferation but is killed by acid secreted by the acid producing tumour cells, $T_\mathrm{A}$. In contrast, tumour cells ($T_\mathrm{A}$ and $T_\mathrm{M}$) are assumed to be resilient to acid. ECM blocks movement of the tumour cells, but can be removed by the matrix-degrading tumour cells, $T_\mathrm{M}$. The two types of tumour cells compete for resources thereby inhibiting each other’s growth (Color figure online)

## The Mathematical Model

Our model builds on the Gatenby–Gawlinski model (Gatenby and Gawlinski 1996) and consists of five components: stroma ($S({\mathbf {x}},t)$), ECM ($M({\mathbf {x}},t)$), a population of acid-producing tumour cells ($T_\mathrm{A}({\mathbf {x}},t)$), lactic acid ($L({\mathbf {x}},t)$), and a population of matrix-degrading tumour cells ($T_\mathrm{M}({\mathbf {x}},t)$), where ${\mathbf {x}}$ denotes space and *t* represents time (Fig. 2). Following Gatenby and Gawlinski (1996), we assume that densities are large enough to be describable by continuous functions and model the spatio-temporal evolution of the system using a combination of spatially distributed ordinary differential equations (ODEs) and PDEs.

### Healthy Tissue Components

We consider two components of healthy tissue: stroma and ECM. The model for the stroma, denoted as *S*, is taken from Gatenby et al. (2006) and assumes that:

- Stromal cells grow logistically at a rate $r_{S}$ and carrying capacity $K_{S}$ in the absence of tumour, reflecting homeostasis.
- Stromal cells are anchored in place, and their motility can be neglected.
- Stromal cells are killed by the lactic acid produced by the tumour cells at a rate proportional to its concentration, $L({\mathbf {x}},t)$, with a constant of proportionality $d_S$.

This yields the following governing equation for $S({\mathbf {x}},t)$:

1 $$\begin{aligned} \frac{\hbox {d} S}{\hbox {d} t} = \overbrace{r_{S} S \left( 1-\frac{S}{K_{S}}\right) }^{\text {Growth}} \quad \overbrace{- d_S L S.}^{\begin{array}{c} \text {Acid} \\ \text {Induced Death} \end{array}} \end{aligned}$$

In modelling the ECM dynamics, we assume:

- There is a net loss of ECM over the time frame of interest. Since we are interested in studying the dynamics of invasion, we will assume that the breakdown of matrix by the tumour overcomes any regeneration, as has been done in other works previously (e.g. Perumpanani and Byrne 1999; Webb et al. 1999; Martin et al. 2010).
- ECM degradation or remodelling is a localised process. This is based on the fact that MDEs are either directly located on the cell membrane or are so large that their diffusion coefficients are very small (Werb 1997). We model this with a linear mass-action model such that ECM is degraded at a rate proportional to the density of matrix-degrading tumour cells, $T_\mathrm{M}({\mathbf {x}},t)$, with $d_\mathrm{M}$ the rate of degradation.
- Because of its fibrous nature, diffusion of ECM can be neglected.

Thus, we model the $M({\mathbf {x}},t)$ dynamics as:

2 $$\begin{aligned} \frac{\hbox {d} M}{\hbox {d} t} = \overbrace{- d_\mathrm{M} T_\mathrm{M} M.}^{\text {Degradation by Tumour}} \end{aligned}$$

Similar models have previously been used in Anderson et al. (2000) and Martin et al. (2010). We remark that both (1)–(2) are ordinary differential equations that are distinct for every spatial point. Furthermore, the equations between neighbouring spatial points are not directly coupled. Instead coupling occurs via one of the other variables (e.g. $T_\mathrm{A}$).

### The Tumour Environment

We consider two phenotypically distinct tumour populations: (i) glycolytic, acid-producing cells ($T_\mathrm{A}({\mathbf {x}},t)$), which release lactic acid killing stromal cells and (ii) matrix-degrading tumour cells ($T_\mathrm{M}({\mathbf {x}},t)$), which degrade the ECM. We assume that:

- In the absence of other cells, each tumour population grows logistically at rates $r_{T_\mathrm{A}}$, $r_{T_\mathrm{M}}$ and carrying capacities $K_{T_\mathrm{A}}$, $K_{T_\mathrm{M}}$, respectively.
- The tumour cells compete with each other and with the stroma for resources and space. We assume competition follows a generalised Lotka–Volterra functional response (Murray 2002), characterised by competition parameters $c_{i,j}$. These describe the inter-species competition that species *j* experiences from species *i* relative to the intra-species competition *i* exerts on itself.
- The tumour cells are motile, but their movement is restricted by the physical obstruction of the ECM. Following Martin et al. (2010), we model obstruction by the ECM as a linear reduction in the flux of cells. We denote by $D_{T_\mathrm{A}}$ and $D_{T_\mathrm{M}}$ the diffusive fluxes in the absence of ECM, and by $K_\mathrm{M}$ the density of ECM such that tumour motility ceases.
- Tumour cells are resilient to the acid. Histology shows adaptation of tumour cells to acidic environments (Gatenby and Gawlinski 1996; Gatenby and Gillies 2004), and theoretical work supports that acid resistance is acquired early in oncogenesis (Robertson-Tessi et al. 2015).

We note that our assumptions about the interactions between the tumour cells and their environment differ to those made by Gatenby and Gawlinski (1996) and Martin et al. (2010), on whose work our study is built. Specifically, Gatenby and Gawlinski choose to neglect competition between tumour and stroma, and Martin et al. include the stroma as an additional physical obstruction to movement. Our choice of assumptions is motivated by the aim to make the two barriers act orthogonally, so to compare their effects. As it appears easier for cells to squeeze through the stroma in a migrating manner than the ECM, we choose this particular order. Determining which assumptions are more physiologically realistic will require further study, but we anticipate that the results presented below will motivate such investigations.

In summary, we propose the following model equations for $T_\mathrm{A}({\mathbf {x}},t)$ and $T_\mathrm{M}({\mathbf {x}},t)$:

3 $$ \begin{aligned} \frac{\partial T_\mathrm{A}}{\partial t}&= \overbrace{r_{T_\mathrm{A}} T_\mathrm{A} \left( 1-\frac{c_\mathrm{S,A} S + T_\mathrm{A} + c_\mathrm{M,A} T_\mathrm{M}}{K_{T_\mathrm{A}}}\right) }^{\text {Growth } \& \text { Competition}}+ \overbrace{\nabla \left[ D_{T_\mathrm{A}} \left( 1 - \frac{M}{K_\mathrm{M}} \right) \nabla T_\mathrm{A} \right] }^{\text {Migration}},\end{aligned}$$

4 $$ \begin{aligned} \frac{\partial T_\mathrm{M}}{\partial t}&= \overbrace{r_{T_\mathrm{M}} T_\mathrm{M} \left( 1-\frac{c_\mathrm{S,M} S + c_\mathrm{A,M} T_\mathrm{A} + T_\mathrm{M}}{K_{T_\mathrm{M}}}\right) }^{\text {Growth } \& \text { Competition}} + \overbrace{\nabla \left[ D_{T_\mathrm{M}} \left( 1 - \frac{M}{K_\mathrm{M}} \right) \nabla T_\mathrm{M} \right] .}^{\text {Migration}} \end{aligned}$$

The final governing equation is that for the acid. We adopt the model by Gatenby and Gawlinski (1996) and assume that:

- The acid is produced by the glycolytic phenotype, $T_\mathrm{A}$, at constant rate $r_L$.
- Acid is removed from the tissue by blood vessels and natural buffering agents at a rate $d_L$. We make the simplifying assumption that this is constant, ignoring possible effects of tumour-induced angiogenesis.
- Because of its small molecular size, acid can diffuse unobstructedly.

This yields the following PDE for $L({\mathbf {x}},t)$:

5 $$\begin{aligned} \frac{\partial L}{\partial t} = \overbrace{r_L T_\mathrm{A}}^{Production} \overbrace{- d_L L}^{Evacuation} + \overbrace{D_L \nabla ^2 L,}^{\text {Diffusion}} \end{aligned}$$

where $r_L$ is the acid production rate, $d_L$ the degradation rate, and $D_L$ the diffusion constant.

### Further Simplifying Assumptions

The aim of this paper is to investigate competition and cooperation between tumour cells based on distinct phenotypic properties. Thus, we will make the simplifying assumption that the two tumour populations are biologically identical, except in their abilities to degrade matrix and produce acid. We will assume identical growth rates, $r_{T_\mathrm{A}} = r_{T_\mathrm{M}} := r_T$, identical carrying capacities (corresponding to intra-species competition), $K_{T_\mathrm{A}} = K_{T_\mathrm{M}} := K_T$, and identical motility, $D_{T_\mathrm{A}} = D_{T_\mathrm{M}} := D_T$. Moreover, we will assume that inhibition received from the stroma is equal for both phenotypes, so that $c_\mathrm{S,A} = c_\mathrm{S,M} := c_S$. Finally, we will also adopt the assumption made by Gatenby and Gawlinski (1996) that stroma and tumour cells have the same carrying capacities $K_T = K_{S} := K$.

We will study the model on a 1-d slice of tissue $\varTheta = [0,\ell ]$, where $x=0$ is the position of the initial core of the tumour and $\ell$ is the length of the slice. We assume that the tumour has initially infiltrated a distance $\sigma < \ell$ which we model by the following initial data:

$$\begin{aligned} S(x,0)&= 1 - f(x; \sigma , \omega ),\\ T_\mathrm{A}(x,0)&= f(x; \sigma , \omega ),\\ T_\mathrm{M}(x,0)&= f(x; \sigma , \omega ),\\ L(x,0)&= f(x; \sigma , \omega ),\\ M(x,0)&= 1 - f(x; \sigma , \omega ), \end{aligned}$$

where $f(\sigma , \omega )$ is a regularised step function and $\omega$, a fixed positive constant, describes the sharpness of the initial boundary between the tumour and the healthy tissue. Specifically:

6 $$\begin{aligned} f(x; \sigma , \omega ) = {\left\{ \begin{array}{ll} 1, &{} \text {if}\ x< \sigma - \omega , \\ \exp \left( 1-\frac{1}{1-(\frac{x - \sigma + \omega }{\omega })^2}\right) , &{} \text {if}\ \sigma - \omega \le x < \sigma ,\\ 0, &{} \text {otherwise.} \end{array}\right. } \end{aligned}$$

To facilitate numerical simulation, we follow previous work (e.g. Gatenby and Gawlinski 1996; Martin et al. 2010) in assuming that there are hard boundaries at $x = 0$ and $x = \ell$, which allows us to close the system with zero-flux boundary conditions (at $x = 0,\ell$). However, as the choice of the domain $\varTheta$ is motivated more by numerical convenience than biological reality, we will only simulate this system for as long as the tumour is far away from the right boundary, to avoid introducing any boundary condition artefacts.

### Non-dimensionalisation

We introduce the following scalings, adopted from Gatenby and Gawlinski (1996) and motivated by the natural scales present in the system:

7 $$\begin{aligned} \tilde{S} = \frac{S}{K}, \;&\tilde{T}_\mathrm{A} = \frac{T_\mathrm{A}}{K}, \; \tilde{T}_\mathrm{M} = \frac{T_\mathrm{M}}{K}, \; \tilde{L} = \frac{L d_L}{r_L K}, \; \tilde{M} = \frac{M}{K_\mathrm{M}},\nonumber \\&\tilde{t} = r_{S} t \; \text {and} \; \tilde{x} = \sqrt{\frac{r_{S}}{D_L}} x. \end{aligned}$$

Based on the parameters used in this study (see also Sect. 2.5), this corresponds to a time scale of 11.57 days and a spatial scale of 2.24 cm. Following previous work (Gatenby and Gawlinski 1996; Martin et al. 2010; McGillen et al. 2014), we choose $\ell$ such that $\tilde{x}$ ranges from 0 to 1 for convenience. Preliminary simulations showed that this allows us to simulate for a time frame of $>600$ days for most parameter combinations before the tumour starts interfering with the right boundary, which is a clinically realistic time scale (equivalent to 1 cm of tumour growth).

Dropping the $\, \tilde{} \;$ for notational convenience, the rescaled model reads:

8 $$\begin{aligned} \frac{\partial S}{\partial t}&= S (1- S) - \delta S L, \end{aligned}$$

9 $$\begin{aligned} \frac{\partial T_\mathrm{A}}{\partial t}&= \rho _T \, T_\mathrm{A} (1 - c_S S - T_\mathrm{A} - c_\mathrm{M,A} T_\mathrm{M}) + \varDelta _T \nabla _x \cdot [ \left( 1 - M\right) \nabla _x T_\mathrm{A}], \end{aligned}$$

10 $$\begin{aligned} \frac{\partial T_\mathrm{M}}{\partial t}&= \rho _T \, T_\mathrm{M} (1 - c_S S - c_\mathrm{A,M} T_\mathrm{A} - T_\mathrm{M}) + \varDelta _T \nabla _x \cdot [\left( 1 - M\right) \nabla _x T_\mathrm{M}], \end{aligned}$$

11 $$\begin{aligned} \frac{\partial L}{\partial t}&= \rho _L (T_\mathrm{A} - L) + \nabla ^2 L, \end{aligned}$$

12 $$\begin{aligned} \frac{\partial M}{\partial t}&= - \kappa T_\mathrm{M} M, \end{aligned}$$

where the dimensionless parameters are given by:

$$\begin{aligned} \delta = \frac{d_S r_L}{d_L r_{S}} K, \quad \rho _T = \frac{r_T}{r_{S}}, \quad \varDelta _T = \frac{D_T}{D_L}, \quad \rho _L = \frac{d_L}{r_{S}}, \quad \kappa = \frac{d_M K}{r_{S}}. \end{aligned}$$

### Parameters

**Table 1:** Parameters used in the numerical simulation of the model

Parameter	Value	References
$\delta$	12.5	Gatenby and Gawlinski (1996)
$\rho _T$	1	Gatenby and Gawlinski (1996)
$\varDelta _T$	$4 \times 10^{-5}$	Gatenby and Gawlinski (1996)
$\rho _L$	70	Gatenby and Gawlinski (1996)
$\kappa$	10	Anderson et al. (2000)
$c_{S}$	1.5	Estimated
$c_\mathrm{M,A}$	0–2	Estimated
$c_\mathrm{A,M}$	0–2	Estimated

As far as possible, we take parameters obtained from the literature. A summary of all the parameters is shown in Table 1. The value for $\kappa$ was adapted from Anderson et al. (2000) where it represents the maximum rate at which the MDEs can degrade the ECM. We carry out parameter sweeps in the competition parameters, as these are difficult to estimate from existing data. As we will see, the choices of ranges for competition parameters encapsulate all of the behaviours we would expect from such a model, and simulations outside these ranges can be inferred from our results. Finally, since we are interested in the ecological interaction of the two phenotypes, not their evolutionary history, we do not consider evolution and hold all parameters constant throughout each simulation.

## Steady State Analysis

During invasion, tumour cells arrive in healthy tissue and establish a self-sustaining population. In principle, this corresponds to a travelling wave solution (TWS) to Eqs. (8)–(12) which connects two spatially homogeneous steady states: the state $(S, T_\mathrm{A}, T_\mathrm{M}, L, M) = (1, 0, 0, 0, M^*)$, where $M^*$ is an arbitrary level of the matrix density (henceforth referred to as SS0), representing healthy tissue and another spatially homogeneous state $(S, T_\mathrm{A}, T_\mathrm{M}, L, M)$ which describes the composition of the invaded tissue. Neglecting the trivial steady state where all cell populations are extinct, the system admits six further steady states:

- *SS 1*$(S, T_\mathrm{A}, T_\mathrm{M}, L, M) = (0, 1, 0, 1, M^*)$, which represents a tumour composed only of acid-producing cells, $T_\mathrm{A}$.
- *SS 2*$(S, T_\mathrm{A}, T_\mathrm{M}, L, M) = (0, 0, 1, 0, 0)$, which describes a tumour composed only of matrix-degrading cells, $T_\mathrm{M}$.
- *SS 3*$(S, T_\mathrm{A}, T_\mathrm{M}, L, M) = (0, \frac{1-c_\mathrm{M,A}}{1-c_\mathrm{M,A} c_\mathrm{A,M}}, \frac{1-c_\mathrm{M,A}}{1-c_\mathrm{M,A} c_\mathrm{A,M}}, \frac{1-c_\mathrm{M,A}}{1-c_\mathrm{M,A} c_\mathrm{A,M}}, 0)$, which describes cancerous tissue in which $T_\mathrm{A}$ and $T_\mathrm{M}$ coexist. Both the stroma and the ECM have been eradicated.
- *SS 4*$(S, T_\mathrm{A}, T_\mathrm{M}, L, M) = (\frac{1-\delta }{1-c_S \delta }, \frac{1-c_S}{1-c_S \delta }, 0, \frac{1-c_S}{1-c_S \delta }, M^*)$, which models a tumour composed of a mixture of acid-producing cells, stroma and ECM.
- *SS 5*$(S, T_\mathrm{A}, T_\mathrm{M}, L, M) = (1, 0, 1-c_S, 0, 0)$, which is representative of a tumour consisting of a mixture of matrix-degrading cells and stroma. As we assume that $c_S>1$, this state is never feasible and so not relevant to this study.
- *SS 6* If $(c_\mathrm{M,A},c_\mathrm{A,M})=(1,1)$, $(S, T_\mathrm{A}, T_\mathrm{M}, L, M) = (1-\delta T_\mathrm{A}, T_\mathrm{A}, 1-c_S-(1-c_S \delta ) T_\mathrm{A}, T_\mathrm{A}, 0),$ where $T_\mathrm{A} \in (0,1)$. Otherwise, $(S, T_\mathrm{A}, T_\mathrm{M}, L, M) = (\frac{1-\delta -c_\mathrm{A,M} c_\mathrm{M,A} +c_\mathrm{M,A} \delta }{1-c_S \delta -c_\mathrm{A,M} c_\mathrm{M,A} +c_\mathrm{M,A} c_S \delta }, \frac{1-c_S-c_\mathrm{M,A}+c_\mathrm{M,A} c_S}{1-c_S \delta -c_\mathrm{A,M} c_\mathrm{M,A} +c_\mathrm{M,A} c_S \delta }, \frac{1-c_S-c_\mathrm{A,M}+c_\mathrm{A,M} c_S}{1-c_S \delta -c_\mathrm{A,M} c_\mathrm{M,A} +c_\mathrm{M,A} c_S \delta }$,
  $\frac{1-c_S-c_\mathrm{M,A}+c_\mathrm{M,A} c_S}{1-c_S \delta -c_\mathrm{A,M} c_\mathrm{M,A} +c_\mathrm{M,A} c_S \delta }, 0)$. As such, SS6 represents acidic cancerous tissue in which all three cell populations coexist. The matrix has been degraded.

**Fig. 3:**
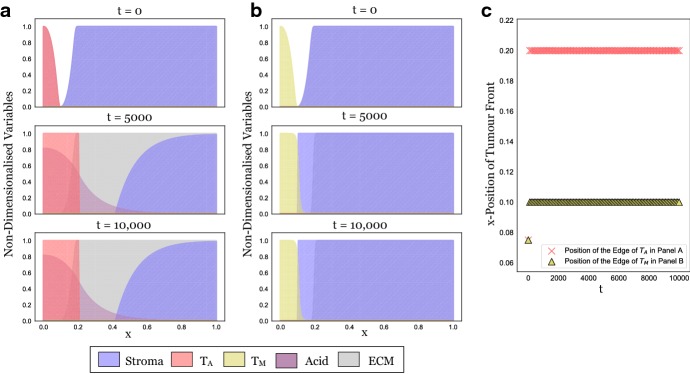
In isolation, the tumour populations fail to invade. **a** Snapshots at three time points from a long-term simulation ($t_{\text {end}} = 10{,}000$, corresponding to more than 10 years) in which only $T_\mathrm{A}$ is present. Expansion stalls because of obstruction by the matrix. **b** Analogous simulation of the dynamics with $T_\mathrm{M}$ in isolation. This time the tumour cannot overcome the stroma. **c** Plot showing the position of the tumour edge in Panels A and B over time, determined as $\min _{x \in [0,1]} \left\{ \hbox {d}/\hbox {d} t (T_i (t)) \right\}$ for $i=\text {A},\text {M}$, respectively. We conclude that the model and the numerical scheme behave as expected and that any invasion seen later in this paper is due to the interaction between the two cell types (Colour figure online)

A linear stability analysis shows that all steady states involving a nonzero density of ECM (SS1 and SS4) have a zero eigenvalue (for details see “Appendix A2”). This corresponds to a perturbation in the ECM density and reflects the fact that, in the absence of $T_\mathrm{M}$, the ECM density will remain constant and all values for *M* are admissible as steady states. Furthermore, we find that SS2, SS4, and SS6 always have at least one eigenvalue with positive real part for the range of parameters considered (Fig. 7). This implies that these states cannot be part of a TWS representing an invading tumour. In contrast, SS1 is linearly stable if $c_\mathrm{A,M}>1$, whereas SS3 is stable if $c_\mathrm{A,M}, c_\mathrm{M,A}<1$ (assuming $\delta >1$; “Appendix A2”). We conclude that there are four possible scenarios for invasion:

1. *Stable Coexistence*: If $c_\mathrm{A,M}<1$ and $c_\mathrm{M,A}<1$, then both tumour populations stably coexist inside the tumour (SS3), resulting in an invading tumour corresponding to a TWS connecting SS3 and SS0.
2. *Competitive Exclusion of*$T_\mathrm{A}$: If $c_\mathrm{A,M}<1$ and $c_\mathrm{M,A}>1$, then $T_\mathrm{M}$ drives $T_\mathrm{A}$ to extinction inside the tumour. Where stroma is present, the healthy tissue is restored (SS0); where it is absent, the system settles into a monoculture of $T_\mathrm{M}$ (SS2). The tumour becomes encapsulated, and invasion halts.
3. *Competitive Exclusion of*$T_\mathrm{M}$: If $c_\mathrm{A,M}>1$ and $c_\mathrm{M,A}<1$, then $T_\mathrm{A}$ drives $T_\mathrm{M}$ to extinction inside the tumour (SS1). While this might nevertheless give rise to a TWS, we conjecture that the associated speed of invasion is zero due to the obstruction from the matrix. We provide numerical evidence for this in Fig. 3.
4. *Bi-Stability*:  If $c_\mathrm{A,M}>1$ and $c_\mathrm{M,A}>1$, then the system is bi-stable and the outcome of invasion is dependent on the initial conditions. We explore this case numerically in Sect. 4.2.3.

## Numerical Simulations

We simulate our model using the method of lines by discretising space, and then applying a standard ODE integration scheme in time. We discretise the equations in space using the following central difference scheme:

$$\begin{aligned} \frac{\partial }{\partial x} \left( D \frac{\partial u}{\partial x} \right) \bigg |_r \approx&\frac{1}{2 h^2} \left( \left( D\big |_{r-1} + D\big |_{r} \right) u_{r-1} - \left( D\big |_{r-1} + 2 D\big |_{r} + D\big |_{r+1} \right) u_r \right. \\&+\left. \left( D\big |_{r} + D\big |_{r+1} \right) u_{r+1} \right) , \end{aligned}$$

where $\big |_r$ denotes evaluation at the $r\text {th}$ spatial grid point, $x_r$, of an equi-spaced grid with grid size *h*, and $D = 1-M$ for the tumour phenotypes, and $D = 1$ for the acid. In the case of the standard Laplacian operator [as in (11)], this reduces to the standard three-point stencil, whereas for (9) and (10), it provides a consistent discretisation of the nonlinear diffusive flux due to the presence of the matrix *M*. The resulting system of ODEs is solved with backwards differentiation formulas (BDF1-BDF5) (Süli and Mayers 2003) implemented in Scipy (specifically, the scipy.integrate.ode class). To improve numerical stability, a stabilisation scheme is used to guide state variables back to zero should they become negative (for details see the provided code). Convergence in space and time for this scheme was checked thoroughly (not shown). The solutions presented are at a resolution of $\delta x = 5 \times 10^{-3}$ in space (200 equally spaced points) and relative, and absolute numerical tolerances of $1 \times 10^{-10}$ were used for the solution in time. Unless otherwise stated, simulations were run until time $t=50$ (corresponding to around 575 days). All simulations were carried out in Python 3.6, using Scipy 1.1.0 and Numpy 1.15.1. Visualisations were produced with Pandas 0.23.4, Matplotlib 2.2.3, and Seaborn 0.9.0. The code is available at: https://github.com/ms234/CooperationInCancerInvasion.

### Neither Acid-Producing Nor Matrix-Degrading Tumour Cells Invade in Isolation

In Fig. 3, we show model simulations in which only one of the two populations is present. We see that in isolation, neither $T_\mathrm{A}$ nor $T_\mathrm{M}$ can invade. In accordance with the linear analysis in Sect. 3, we see that if only $T_\mathrm{A}$ is present, then the tumour initially advances, but invasion halts because of obstruction by the matrix (Fig. 3a). Similarly, if only $T_\mathrm{M}$ is present, then the tumour is encapsulated by the stroma (Fig. 3b). Plotting the position of the tumour edge in each case confirms this (Fig. 3c).

**Fig. 4:**
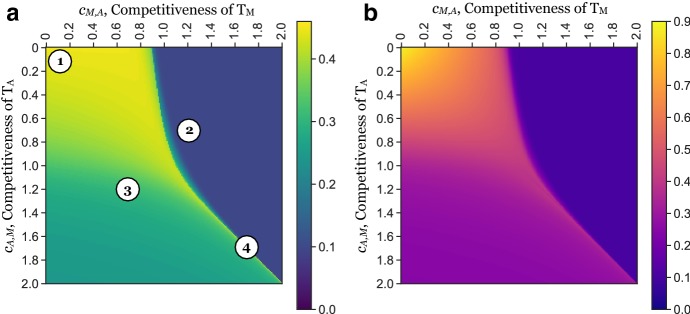
The invasive potential of a tumour is determined by the competition between its subpopulations. **a** Position of the tumour front at time $t = 50$ (575 days) as a function of the strength of inter-species competition ($c_\mathrm{M,A}$ and $c_\mathrm{A,M}$). This was defined as $\max (x_\mathrm{A}, x_\mathrm{M})$, where $x_i = \min _{x \in [0,1]} \left\{ \hbox {d}/\hbox {d} t (T_i (50)) \right\}$ for $i=$ A or M, respectively, is the position of the wave front of $T_\mathrm{A}$ and $T_\mathrm{M}$ at time 50. Annotations (numbers in circles) correspond to the time-series plots as shown in Fig. 5. We find the tumour advances furthest when inter-species competition is weaker than intra-species competition ($c_\mathrm{M,A}, c_\mathrm{A,M} < 1$). As the strength of inter-species competition increases above that of intra-species competition ($c_\mathrm{M,A}, c_\mathrm{A,M} > 1$), invasion slows, especially if $T_\mathrm{M}$ dominates. **b** Total tumour mass, defined as ${\mathcal {M}} = \int _{x=0}^1 T_\mathrm{A}(x,50) + T_\mathrm{M}(x,50) \hbox {d}x$, as a function of the inter-species competition. We see that the total tumour mass in the invading tumour, which may be interpreted as a proxy for the total cell number, is a strictly and rapidly decreasing function of the competition parameters. Thus, competition between tumour cells influences not only how far they invade, but also how many cells make up the advancing tumour. *Note*: cases in which the cell populations were small ($\int _{x=0}^{1} T_i(x, 50) \hbox {d}x < 0.1$) were disregarded in this analysis to avoid issues associated with the simulation and interpretation of low densities (Colour figure online)

**Fig. 5:**
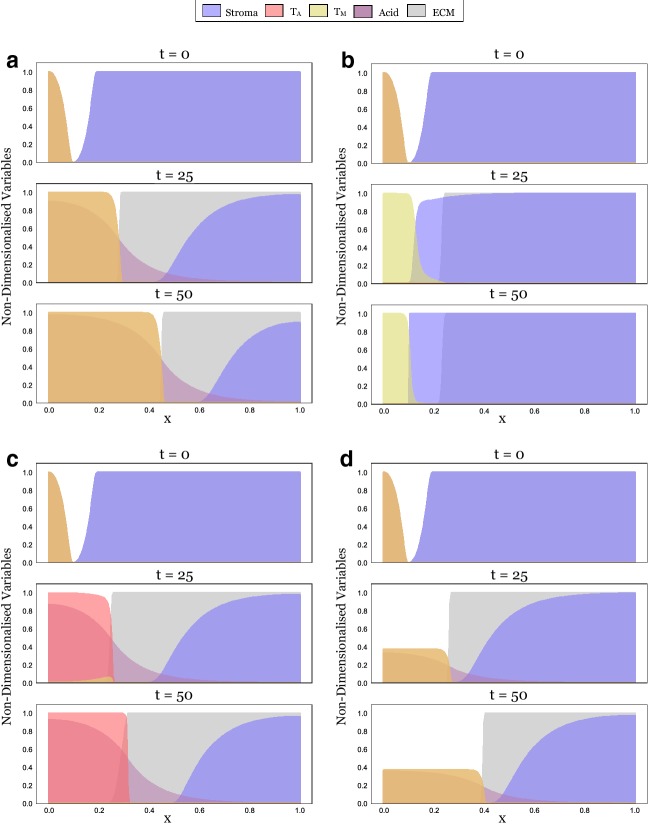
Simulations illustrating the four different scenarios that can occur depending on the inter-species competition between $$T_\mathrm{A}$$ and $$T_\mathrm{M}$$. Panels correspond to the locations in the competition parameter space marked in Fig. 4a (1:A, 2:B, 3:C, 4:D). **a**$$(c_\mathrm{M,A},c_\mathrm{A,M}) = (0,0)$$. At $$t=0$$, the tumour begins as a mixture of acid-producing (red) and matrix-degrading cells (yellow) on the left-hand side of the domain (appearing orange due to the mixture of the colours). It is constrained by a mixture of stroma (blue) and ECM (grey) on the right-hand side (appearing as dark blue). Since inter-species competition is weak, the tumour populations can coexist and combine their traits, allowing them to invade rapidly ($$t=25$$ and $$ t=50$$). **b**$$(c_\mathrm{M,A},c_\mathrm{A,M}) = (1.2, 0.7)$$. In contrast, when $$T_\mathrm{M}$$ dominates over $$T_\mathrm{A}$$, it drives $$T_\mathrm{A}$$ to extinction and no invasion takes place. **c**$$(c_\mathrm{M,A},c_\mathrm{A,M}) = (0.7,1.2)$$. $$T_\mathrm{A}$$ dominates over $$T_\mathrm{M}$$. While invasion eventually stops due to a lack of ECM degradation, the tumour initially invades thanks to a small population of $$T_\mathrm{M}$$ persisting at the tumour edge (appearing in orange at $$t=25$$). **d**$$(c_\mathrm{M,A},c_\mathrm{A,M}) = (1.7, 1.7)$$. Mutual exclusion of $$T_\mathrm{A}$$ and $$T_\mathrm{M}$$. When seeded at equal densities, the two populations will invade as shown, but the invading front is not stable. If a small perturbation is introduced, the two populations will separate and invasion will halt (Fig. 8)

### Intra-tumoural Competition Determines the Tumour’s Invasion Properties

Our results in Sect. 3 show that when both tumour cell populations are present, there are four different possible outcomes depending on the strength of the inter-species competition between $T_\mathrm{A}$ and $T_\mathrm{M}$. To further investigate this relationship, we simulated invasion for $10^4$ combinations of values of $(c_\mathrm{M,A},c_\mathrm{A,M})$ equally spaced on the grid $[0, 2] \times [0, 2]$, corresponding to rates of inter-species competition between zerofold and twofold that of the intra-species competition. We initialised the tumour as described in Sect. 2.3 with $\omega = 0.1, \sigma = 0.2$ for *S* and $M, \sigma = 0.1$ for $T_A$, $T_M$ and *L*, and simulated until time $t = 50$.

#### Stable Coexistence of Multiple Tumour Phenotypes Promotes Invasion

Figure 4a shows the position of the tumour edge at $t = 50$ for these $10^4$ parameter combinations. We find that the tumour invades furthest for $c_\mathrm{M,A}, c_\mathrm{A,M} < 1$, corresponding to the case when inter-species competition is weaker than intra-species competition. Studying the solution for $(c_\mathrm{M,A}, c_\mathrm{A,M}) = (0,0)$ shows that for this range of values, the two populations mix and advance as a single front (Fig. 5a).

Furthermore, we observe that the relationship between the invaded distance and the competition parameters is not symmetric about $c_\mathrm{M,A}$ and $c_\mathrm{A,M}$. In particular, provided $c_\mathrm{M,A}, c_\mathrm{A,M} < 1$, the invaded distance is more sensitive to a higher competitiveness of $T_\mathrm{A}$ than $T_\mathrm{M}$. Repeating the experiment in Fig. 4a with different rates of matrix degradation, $\kappa$, shows that this asymmetry is due to $\kappa$ (“Appendix A3”). For the parameters shown in Fig. 4a, matrix remodelling is less effective than removal of the stroma for the parameters shown, essentially creating a bottleneck. Our results in Sect. 3 show that a larger ratio of $c_\mathrm{M,A}$ to $c_\mathrm{A,M}$ corresponds to a larger proportion of $T_\mathrm{M}$ in steady state allowing for more matrix degradation to take place. To summarise, we find that the most invasive tumours are those in which $T_\mathrm{A}$ and $T_\mathrm{M}$ mix and locally coexist in the correct proportions.

#### Competitive Exclusion Slows Tumour Invasion

As $c_\mathrm{M,A}$ is increased through 1, so that $c_\mathrm{M,A}>1$ and $c_\mathrm{A,M} < 1$, we observe a rapid reduction in tumour expansion (Fig. 4a). A simulation for $(c_\mathrm{M,A}, c_\mathrm{A,M}) = (1.2,0.7)$ shows that in this domain, $T_\mathrm{M}$ drives $T_\mathrm{A}$ to extinction inside the tumour and is subsequently encapsulated by the stroma due to a lack of acid to keep the stroma in check (Fig. 5b).

Similarly, if $T_\mathrm{A}$ out-competes $T_\mathrm{M}$ ($c_\mathrm{M,A}<1$ and $c_\mathrm{A,M}>1$), then invasion is also reduced (Fig. 4a). However, this reduction is less significant than in the converse case. This is because the $T_\mathrm{M}$ population transiently survives near the edge of the tumour ($t = 25$ in Fig. 5c), where it degrades the ECM for the advancing bulk of the tumour until it is eventually eradicated ($t = 50$ in Figs. 5c, 10).

#### Strong Inter-species Competition Prevents Clonal Mixing and Reduces Invasion

When inter-species competition is stronger than intra-species competition for both populations ($c_\mathrm{M,A}, c_\mathrm{A,M} > 1$), we observe three possible outcomes: (i) the two populations coexist and invade ($c_\mathrm{M,A} = c_\mathrm{A,M}$ in Fig. 4a), (ii) $T_\mathrm{A}$ out-competes $T_\mathrm{M}$, and the tumour advances only temporarily ($c_\mathrm{M,A} < c_\mathrm{A,M}$ in Fig. 4a), and (iii) $T_\mathrm{M}$ out-competes $T_\mathrm{A}$, and invasion rapidly halts ($c_\mathrm{M,A} > c_\mathrm{A,M}$ in Fig. 4a).

When invasion does occur ($c_\mathrm{M,A} = c_\mathrm{A,M}$), the tumour is also a mixture of the two phenotypes (Fig. 5d); however, the advancing front is unstable to small perturbations (Fig. 8). Similarly, if the two populations are not initialised identically, but placed slightly apart, then they separate spatially (Fig. 8b). Moreover, the solution is strongly sensitive to the parameters, with slight perturbations generating qualitatively different outcomes from the same initial conditions (Fig. 8c, d). In summary, this indicates that cooperation in this regime is unstable, and most likely competitive exclusion or spatially separated populations (parapatry) would be observed.

Formally speaking, solutions along the line in the $(c_\mathrm{M,A},c_\mathrm{A,M})$ parameter space are structurally unstable, corresponding to a separatrix between competitive exclusion of each species. Specifically, away from the invasion front, the stroma, matrix, and acid can be neglected, and the system is simply two Lotka–Volterra-type equations with identical parameters. Neglecting the spatial dynamics and considering the phase-plane of such a system, we see that the stable manifold of the coexistence steady state (which is a saddle) forms a separatrix between the single-species equilibria (see, for instance, Chapter 3 of Murray 2002). This implies that any asymmetry in the initial condition between these two species will lead to one or the other species becoming extinct. Spatial dynamics can then lead to a stabilization of local equilibria of each species, but not to any kind of homogeneous coexistence equilibria, and the spatial structure of the populations can depend sensitively on the initial data. We remark that this separatrix exists even for distinct competition parameters, but for comparable initial densities we do not observe coexistence.

#### The Ratio of Invaded Distance to Tumour Mass Reflects Tumour Ecology

In addition to the distance the tumour has invaded, another important feature in the clinic is the total tumour mass that has developed. We compute this as ${\mathcal {M}} = \int _{x=0}^1 T_\mathrm{A}(x,50) + T_\mathrm{M}(x,50) \hbox {d}x$ and present the results in Fig. 4b. This shows that the two measures are not identical. While the progress of the front is almost identical along the line $(c_\mathrm{M,A}, c_\mathrm{A,M}) = (s, 0)$ for $s \in [0,1]$ (Fig. 4a), the mass of the resulting tumour decreases rapidly (Fig. 4b). A similar pattern holds true along the line $c_\mathrm{M,A} = c_\mathrm{A,M}$ and suggests that the strength of competition between tumour subpopulations affects not only the speed of invasion, but also the density of the resulting tumour mass.

**Fig. 6:**
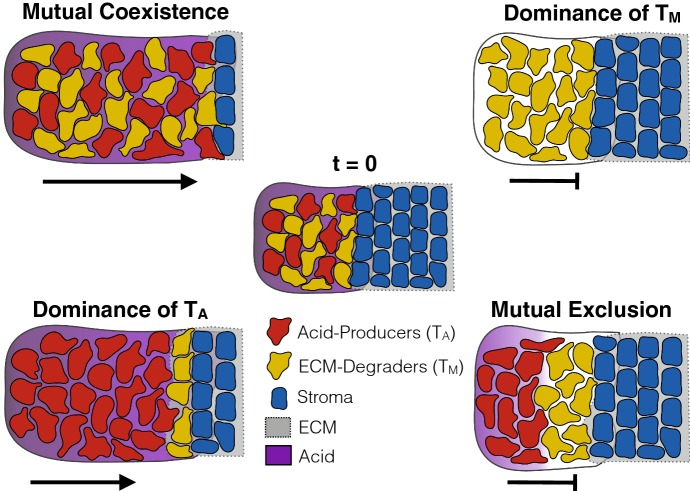
Summary of the key findings of this paper. If the two phenotypes can coexist, a highly invasive community of cells emerges. Conversely, if $T_\mathrm{M}$ dominates, tumour invasion comes to a halt as the cells are unable to overcome the stroma. If $T_\mathrm{A}$ dominates, then a temporarily invasive tumour mass forms in which $T_\mathrm{M}$ cells find a temporary habitat in the matrix at the tumour edge. Finally, in the case where the two cell types mutually exclude each other’s growth, the cells separate into spatially distinct regions and fail to invade (Colour figure online)

## Discussion

While tumour heterogeneity is now widely recognised (Merlo et al. 2006; Gerlinger et al. 2012; Anderson and Maini 2018), we are only beginning to comprehend its implications for cancer progression. The fact that the cells in a tumour are not identical, and instead might act as a collective composed of phenotypically distinct individuals, is particularly important in the context of cancer invasion. Invasion of tissue requires both the ability to degrade or remodel the ECM and the ability to remove surrounding stromal cells. While over time it is possible for the necessary genetic changes to all accumulate in one tumour cell type, it seems more likely that these abilities initially arise in separate cells. Here, we aimed to investigate whether cooperation between distinct phenotypic populations is a viable mechanism for invasion, and to characterise the dynamics of such cooperative invasion.

We summarise our findings in Fig. 6. Our theoretical results show that cooperation between two cell types gives rise to an invading tumour at clinically realistic speeds (1–2 cm in a year). Further, we identify two possible modes of invasion: firstly, when the two cell types compete weakly with each other, allowing both to stably coexist. Our model predicts that the resulting mutualistic community has strong invasive potential, as all required traits are present in the same place at the same time (Fig. 5a). It has previously been observed that tumours with high degrees of clonal mixing are more aggressive (Sottoriva et al. 2015; Zhang et al. 2018). This has so far been explained by higher cell motility and resulting invasive potential (Sottoriva et al. 2015). Based on our results, we propose that an additional explanation could be that mixing allows individual cells to more readily share their traits. As such, we advocate further research into the clinical importance of clonal mixing.

In addition, our model predicts a second mode of invasion, in which the acid-producing cells drive the matrix-degrading cells to extinction throughout the tumour, but can temporarily invade as a population of matrix-degrading cells transiently survives near the edge of the tumour (Fig. 6). While invasion in this case is only transient, it could be a contributing factor to cancer invasion, since further mutations could develop or blood vessels could be reached that would allow for continued growth. Current literature suggests that acid-producing cells would have a competitive advantage over matrix-degrading cells since they are better adapted to low pH conditions (Gatenby and Gillies 2004; Gatenby et al. 2006, 2007), and that the onset of invasion is marked by the expansion of a highly glycolytic cancer phenotype (Robertson-Tessi et al. 2015). Our results indicate that commensualistic or parasitic relationships might develop between aggressive glycolytic cells in the core of the tumour and cells at the tumour edge which might facilitate invasion. Mathematically, our work also illustrates recent results showing that if the dominant species in a diffusive Lotka–Volterra system moves at a slower rate, then the two species invade empty space as a “propagating-terrace”, where the weaker species invades first but is subsequently eradicated by the dominant species (Carrère 2018).

Although it was not our primary objective, our work also highlights the differences between physical and biological barriers to tumour invasion. In our model, the ECM was a purely physical barrier, whereas the stroma acted by suppressing tumour growth. Figure 3 shows that the biological barrier of the stroma is more effective in blocking tumour invasion than the “wall” of ECM. Unless the level of the ECM is precisely 1, $T_\mathrm{A}$ can invade even in the absence of matrix degrading activity and advances until $x=0.3$ (Fig. 3a). In contrast, $T_\mathrm{M}$ is stopped at $x=0.2$ because the arriving tumour cells fail to establish a locally self-sustaining population due to the growth inhibition by the stroma (Fig. 3b). This makes the point that a key challenge for invading tumour cells is to survive and grow in this new environment. Furthermore, we found in modelling obstruction that there remains a number of unsolved mathematical challenges: (i) how do the travelling wave solutions to this nonlinear diffusion model of movement obstruction develop [Eqs. (10) and (12)]? (ii) How do these compare with alternative models of a hard boundary, such as a moving boundary (Du and Guo 2012; El-Hachem et al. 2019)? (iii) How should one model distinct, yet simultaneously acting, physical obstructions?

We note that there are a number of potentially important interactions not accounted for in the model. Firstly, we do not model matrix regeneration (e.g. Martin et al. 2010). It seems plausible that matrix regeneration might make it significantly more challenging for the matrix-degrading cells to invade. As a result, the invasive capabilities of a tumour with a “pocket” of matrix degrading cells might be much smaller than predicted by our model. Conversely, as we discussed in the introduction, some MDEs generate by-products which can stimulate movement of the cells. Anderson et al. (2000) found that this can result in the leading edge of the tumour separating from the main mass. In our model, this might allow the matrix-degrading cells to penetrate further into the tissue and increase invasiveness. Finally, the ECM is composed of proteins and, as such is also subject to acid degradation (McKinnell 1998). Because the aim of this paper was to acquire a first understanding of what general behaviours might emerge, we neglected this degradation in our model. However, clearly, this will influence the invasive behaviour and it would be important to include such a term in future models. Finally, we remark that our approach focused on understanding the invasive front itself, using a simplified model of phenotype interaction (direct competition). It is now well known that the selection pressures at the edge of an invasive front are different from within an organism’s “home range” due to a range of differences near an invading front (the Allee and Olympic Village effects, for instance) (Keymer and Marquet 2014; Perkins et al. 2016; Erm and Phillips 2018; Calvez et al. 2018). More generally, evolution and life history can have strong impacts on dispersal efficiency and range expansion (Benichou et al. 2012; Bouin et al. 2012; Perkins et al. 2013). Investigating these different modes of selection could provide insight into phenotypic heterogeneity throughout a tumour compared to its invading edge.

To sum up, we have explored cooperation of tumour cells as a mode of tumour invasion. We found that the most invasive tumour emerges when cells coexist in the same region in space as this allows cells to most effectively share their traits. This point is simple but important: to fully understand the implications of tumour heterogeneity, we have to ask not only *what cells are present* but also *where are these cells located*? Do they live in separate regions or can they spatially *mix* and, thus, *match* their traits?

## Figures and Tables

**Fig. 7:**
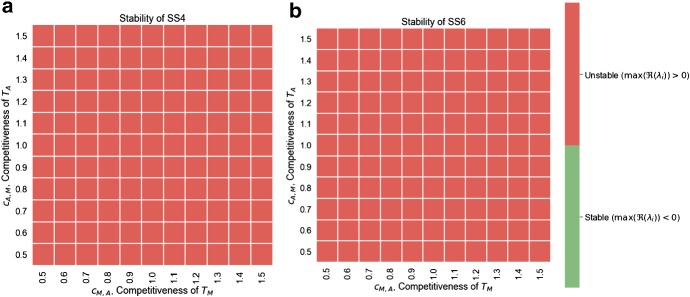
Numerical stability analysis of SS4 (**a**) and SS6 (**b**) for the range of parameters considered in this paper (see Table 1). Stability was assessed by computing the eigenvalues of the Jacobian at the steady state and assessing whether at least one eigenvalue had a strictly positive real part. We find that both SS4 and SS6 are unstable across the range of parameters considered (Color figure online)

**Fig. 8:**
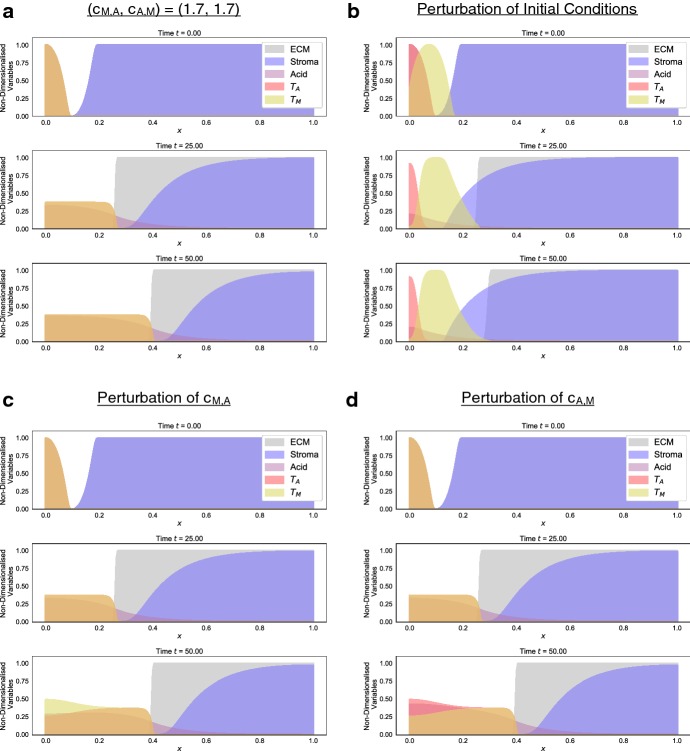
Instability of the invasive front when $c_\mathrm{M,A}=c_\mathrm{A,M}$. **a** Simulation for $c_\mathrm{M,A}=c_\mathrm{A,M}=1.7$. **b** Perturbation of the initial conditions in **a**. The initial distribution of $T_\mathrm{M}$ was shifted by $\delta x=0.15$ to the right, resulting in subsequent spatial separation of the two phenotypes and arresting of invasion. **c** Small perturbation in $c_\mathrm{M,A}$ ($(c_\mathrm{M,A}, c_\mathrm{A,M})=(1.700001, 1.7)$). **d** Small perturbation in $c_\mathrm{A,M}$ ($(c_\mathrm{M,A}, c_\mathrm{A,M})=(1.7, 1.700001)$). This indicates that the system is structurally unstable in this parameter regime (Colour figure online)

**Fig. 9:**
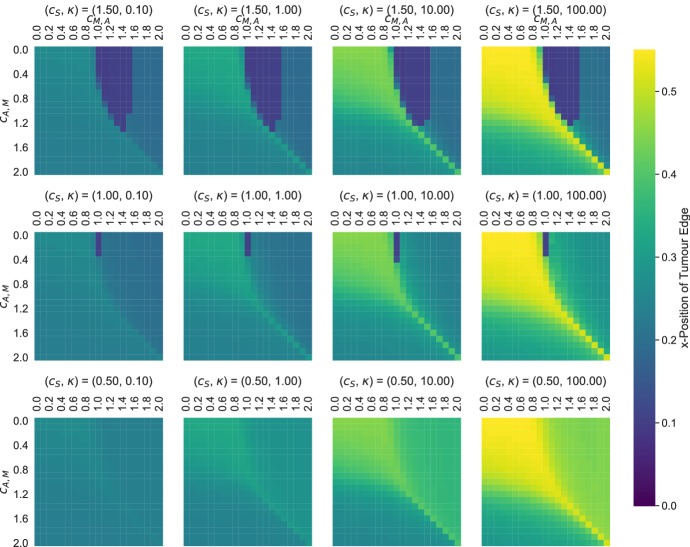
Sensitivity analysis for the parameters $c_\mathrm{M,A}, c_\mathrm{A,M}, c_{S}$, and $\kappa$. Each heatmap shows the position of the front of the tumour at $t = 50$ (computed as for Fig. 4a) (Colour figure online)

**Fig. 10:**
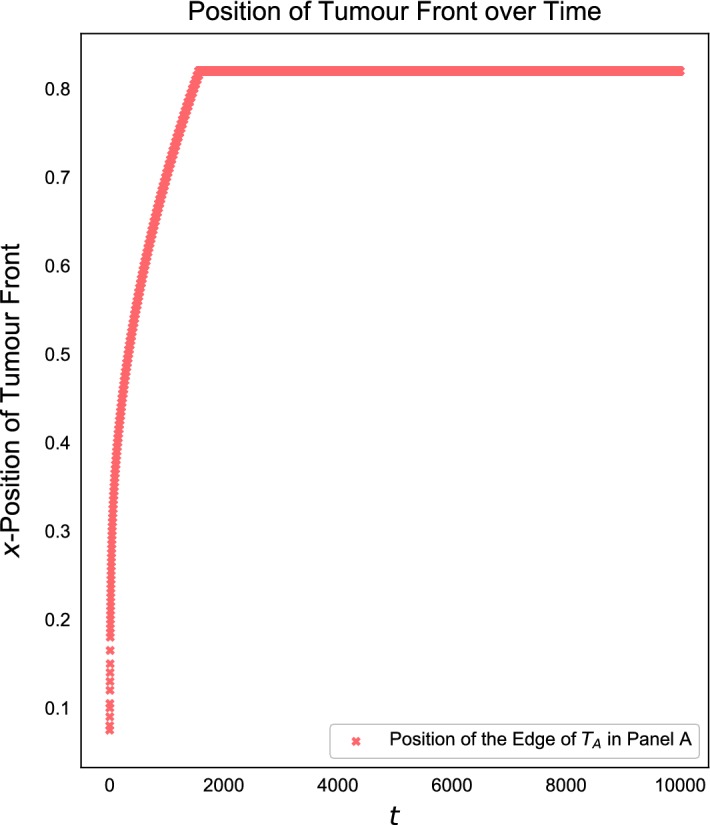
When $T_\mathrm{A}$ drives $T_\mathrm{M}$ to extinction, only transient invasion occurs. Position of the tumour edge simulated until $t=10{,}000$ for $(c_\mathrm{M,A}, c_\mathrm{A,M})=(0.5, 1.5)$. The tumour transiently advances, while the $T_\mathrm{M}$ initially persists. However, eventually $T_\mathrm{M}$ is eradicated and invasion stalls

## Image Collection and Processing

A TMA containing formalin-fixed and paraffin-embedded human breast tissue specimens was constructed at the Moffitt Cancer Center histology core. The TMA contains 27 normal breast tissue, 30 DCIS, 48 invasive ductal carcinomas without metastasis, 49 invasive ductal carcinomas with metastasis, and 48 lymph node macrometastases of breast cancer. Cores were selected from viable tumour regions and did not contain necrosis. A 1:200 dilution of anti-LAMP2b (#ab18529, Abcam) and 1:200 of anti-TGM2 (#ab109200, Abcam) were used as primary antibody. Normal placenta was used as a positive control for LAMP2 and normal human kidney for TGM2. For the negative control, an adjacent section of the same tissue was stained without application of primary antibody, and any stain pattern observed was considered as nonspecific binding of the secondary.

Immunohistochemical analysis was conducted using digitally scanning slides. A pathologist reviewer scored the intensity of each stain on a scale from 0 to 3, where a 0 was considered negative, score 1 was weakly positive, score 2 was moderately positive, and score 3 was strongly positive. For further information, see Damaghi et al. (2019).

In order to create the visualisations in Fig. 1, we extracted masks of only the areas with the highest score (a score of 3). To do so, we first aligned the slides for each stain (LAMP2b and TGM2) using VALIS (in preparation, see also Gatenbee et al. 2019) and subsequently extracted the areas with the highest score using OpenCV. Finally, we overlaid the extracted masks on top of the TGM2 slide (Fig. 7).

**Table 2:** The eigenvalues describing the linear stability of the steady states

State	Description	$\lambda _1$	$\lambda _2$	$\lambda _3$	$\lambda _4$	$\lambda _5$	Stability
SS0	Healthy tissue	0	− 1	$-\rho _L$	$\rho _T (1-c_{S})$	$\rho _T (1-c_{S})$	Yes
SS1	$T_\mathrm{A}$ Monoculture	0	$-\rho _T$	$-\rho _L$	$1-\delta$	${{\varvec{\rho }}_\mathbf{T} (\mathbf{1-c}_{\mathbf{A,M}})}$	If $c_\mathrm{A,M}>1$
SS2	$T_\mathrm{M}$ Monoculture	${\mathbf{1}}$	$-\rho _T$	$-\rho _L$	$-\kappa$	${{\varvec{\rho }}_\mathbf{T}(\mathbf{1-c}_{\mathbf{M,A}})}$	No
SS3	$T_\mathrm{A}-T_\mathrm{M}$ Coexistence	$-\rho _T$	$-\rho _L$	${\frac{{\varvec{\rho }}_\mathbf{T}(\mathbf{1}+\mathbf{c}_{\mathbf{A,M}} \mathbf{c}_{\mathbf{M,A}}-\mathbf{c}_{\mathbf{A,M}}-\mathbf{c}_{\mathbf{M,A}})}{\mathbf{c}_{\mathbf{A,M}} \mathbf{c}_{\mathbf{M,A}}-\mathbf{1}}}$	${\frac{{\varvec{\kappa }}(\mathbf{1-c}_{\mathbf{A,M}})}{\mathbf{c}_{\mathbf{A,M}} \mathbf{c}_{\mathbf{M,A}}-\mathbf{1}}}$	${\frac{{\varvec{\rho }}_\mathbf{T}(\mathbf{1+c}_{\mathbf{M,A}} {\varvec{\delta }}-\mathbf{c}_{\mathbf{A,M}} \mathbf{c}_{\mathbf{M,A}}-{\varvec{\delta }})}{\mathbf{1-c}_{\mathbf{A,M}} \mathbf{c}_{\mathbf{M,A}}}}$	If $c_\mathrm{A,M},c_\mathrm{M,A}<1$
SS4	Stroma-$T_\mathrm{A}$ Coexistence			See Fig. 7			No
SS5	Stroma-$T_\mathrm{M}$ Coexistence	− 1	${{\varvec{\rho }}_\mathbf{L}}$	${{\varvec{\rho }}_\mathbf{T}(\mathbf{c}_\mathbf{S-1})}$	${{\varvec{\kappa }}(\mathbf{c}_\mathbf{S-1})}$	${{\varvec{\rho }}_\mathbf{T}(\mathbf{1+c}_{\mathbf{M,A}}{} \mathbf{c}_\mathbf{S}-\mathbf{c}_\mathbf{S}-\mathbf{c}_{\mathbf{M,A}})}$	No
SS6	Stroma-$T_\mathrm{A}-T_\mathrm{M}$ Coexistence			See Fig. 7			No

Stability is assessed based on the parameter values in Table 1. Bold values indicate eigenvalues that are positive in at least parts of the parameters space. For SS4 and SS6, no simple analytic forms were attainable, and instead stability was assessed numerically (see Fig. 7)

## Stability Analysis

In Table 2, we list the eigenvalues computed for each steady state. For SS4 and SS6, no simple analytic forms were obtainable. Instead, we numerically computed the eigenvalues for the range of parameters of interest and show $\max _{i \in 1,2,3,4,5}(\mathfrak {R}(\lambda _i))$, where $\lambda _i$ denotes the ${i}{\text {th}}$ eigenvalue of the Jacobian (Fig. 7). We conclude that only SS0, SS1, and SS3 are stable. Analytic results were obtained by hand and confirmed with Maple 2018. Numerical computations were carried out in Python 3.6 (for further details on the environment, see Sect. 4) (Fig. 8).

## Sensitivity Analysis for $\kappa$ and $c_S$

As the parameters for the strength of competition between the stroma and the tumour ($c_S$) and the rate of matrix degradation are difficult to obtain experimentally, we performed a sensitivity analysis over the plausible range in which they might lie. In Fig. 9, we show the distance invaded by the tumour, calculated as for Fig. 4. We see that as $\kappa$ is increased, the tumour invades further. Moreover, while for values of $\kappa = 10$ tumours with higher proportion of $T_\mathrm{M}$ (corresponding to lower $c_\mathrm{A,M}$, see also Fig. 4a) appear more aggressive, this is less apparent when the matrix can be degraded faster ($\kappa = 100$) (Figs. 9, 10).
